# Impact of COVID-19 on posttraumatic stress disorder in ICU survivors: a prospective observational comparative cohort study

**DOI:** 10.1186/s13054-024-04826-1

**Published:** 2024-03-14

**Authors:** Pierre Kalfon, Wissam El-Hage, Marie-Agnès Geantot, Constance Favier, Laetitia Bodet-Contentin, Khaldoun Kuteifan, Pierre-Yves Olivier, Didier Thévenin, Julien Pottecher, Jullien Crozon-Clauzel, Bénédicte Mauchien, Arnaud Galbois, Roland de Varax, Sabine Valera, Philippe Estagnasie, Audrey Berric, Martine Nyunga, Nathalie Revel, Georges Simon, Benjamin Kowalski, Achille Sossou, Thomas Signouret, Marc Leone, Charles Delalé, Aurélien Seemann, Sigismond Lasocki, Jean-Pierre Quenot, Antoine Monsel, Olivier Michel, Mathieu Page, René-Gilles Patrigeon, Walid Nicola, Arnaud W. Thille, Guillaume Hekimian, Pascal Auquier, Karine Baumstarck, PTSD-REA Study group, Hortense Catry, Anne-Laure Dubus, Léa Laugery, Marion Lintaff, Mélanie Lourseyre, Lou Merigard, Lisa Michel, Nawal Ouhmad, Solenn Petit, Laurence Tricoche, Florent Beaumale, Anne-Sylvie Scholastique, Emmanuelle Mougenot, Céline Delerue, Marc Feller, Julien Grouille, Charles-Edouard Rochon, Juliette Audibert, Gaëtan Badre, Cécile Jourdain, Leslie Lehaie, Hasni Si Abdelkader, Emilie Henry, Marie Labruyere, Claire Boulle-Geronimi, Stéphanie Beaussard, Olivier Nigeon, Anthea Loiez, Valérie Cerro, Laetitia Marchand, Charlotte Arbelot, Karine Buzelier, Deborah Levy, Pascale Leloup, Karim Messaoudi, Camille Alzina, Lee Nguyen, Steve Nowak, Carole Ichai, Aminata Diop, Hélène Brisson, Jean-Michel Constantin, Samia Lakhal, Madjid Oudihat, Rémi Coudroy, Carole Guyon, Jean-Pierre Frat, René Robert, Nadine Lubango, Lisa Villequey, Stéphane Hecketsweiler, Nicolas Partouche, Laurent Ducros, Vincent Gardan, Julie Rivoire, Stéphanie Deparis-Dusautois, Lamia Lamri, Alexandra Lavalart

**Affiliations:** 1 Réanimation Polyvalente, Hôpital Louis Pasteur, CH de Chartres, Le Coudray, France; 2 Unité de Recherche CEReSS-EA3279, Aix-Marseille Université, Marseille, France; 3 UMR 1253, iBrain, Université de Tours, INSERM, Tours, France; 4 Centre Régional de Psychotraumatologie, CHRU de Tours, Tours, France; 5 Département d’Anesthésie Réanimation, CHU Dijon Bourgogne, Dijon, France; 6 Médecine Intensive Réanimation, INSERM CIC1415, CRICS-TriGGERSep Network, CHRU de Tours, Tours, France; 7 et INSERM UMR1246 SPHERE, Universités de Nantes et Tours, Tours, France; 8 Service de Réanimation Médicale, Groupe Hospitalier de la Région de Mulhouse Sud Alsace, Mulhouse, France; 9 Médecine Intensive Réanimation, CHU d’Angers, Angers, France; 10 Médecine Intensive Réanimation, CH de Lens, Lens, France; 11 Service d’Anesthésie-Réanimation et Médecine Péri-Opératoire, Hôpital Hautepierre, CHU de Strasbourg, Strasbourg, France; 12 Département d’Anesthésie Réanimation, CHU Edouard Herriot, Hospices Civils de Lyon, Lyon, France; 13 Service de Réanimation Polyvalente, Hôpital Privé Claude Galien, Quincy-Sous-Sénart, France; 14 CH de Mâcon, Mâcon, France; 15 Médecine Intensive Réanimation, Hôpital Nord, Assistance Publique–Hôpitaux de Marseille (AP-HM), Marseille, France; 16 Clinique Ambroise Paré, Neuilly-sur-Seine, France; 17 Réanimation Polyvalente, Hôpital Sainte-Musse, Toulon, France; 18 Réanimation Polyvalente, Hôpital Victor Provo, Roubaix, France; 19 Réanimation Médico-Chirurgicale, Hôpital Pasteur, CHU de Nice, Nice, France; 20 CH de Troyes, Troyes, France; 21 CH de Douai, Douai, France; 22 Département d’Anesthésie-Réanimation, Hôpital Émile Roux, Le Puy-en-Velay, France; 23 Hôpital Européen de Marseille, Marseille, France; 24 Réanimation, Département d’Anesthésie-Réanimation, Hôpital Nord, AP-HM, Marseille, France; 25 Réanimation, Hôpital Simone Veil, CH de Blois, Blois, France; 26 Nouvelle Clinique de Tours Saint-Gatien, Tours, France; 27 Réanimation Chirurgicale, CHU d’Angers, Angers, France; 28 Service de Médecine Intensive Réanimation, CHU Dijon Bourgogne, Dijon, France; 29 Département d’Anesthésie-Réanimation, Hôpital Pitié-Salpêtrière, GRC 29, DMU DREAM, Assistance Publique-Hôpitaux de Paris (AP-HP), Sorbonne Université, Paris, France; 30 Service de Réanimation Polyvalente, CH de Bourges, Bourges, France; 31 Clinique Convert, Bourg-en-Bresse, France; 32 Réanimation, CH d’Auxerre, Auxerre, France; 33 CH de Montargis, Montargis, France; 34 Médecine Intensive Réanimation, CHU de Poitiers, Poitiers, France; 35 Service de Médecine Intensive Réanimation, Hôpital Pitié-Salpêtrière, Sorbonne Université AP-HP, Paris, France; 36 Réanimation Polyvalente, Hôpital Privé la Casamance, 33 Boulevard Des Farigoules, 13400 Aubagne, France

**Keywords:** Critical care, Posttraumatic stress disorder, COVID-19, Intensive care unit

## Abstract

**Background:**

Posttraumatic stress disorder (PTSD) after a stay in the intensive care unit (ICU) can affect one in five ICU survivors. At the beginning of the coronavirus disease 2019 (COVID-19) pandemic, admission to the ICU for COVID-19 was stressful due to the severity of this disease. This study assessed whether admission to the ICU for COVID-19 was associated with a higher prevalence of PTSD compared with other causes of ICU admission after adjustment for pre-ICU psychological factors.

**Methods:**

This prospective observational comparative cohort study included 31 ICUs. Eligible patients were adult ICU survivors hospitalized during the first wave of COVID-19 pandemic in France, regardless of the reason for admission. The prevalence of presumptive diagnosis of PTSD at 6 months was assessed using the PTSD Checklist for DSM-5 (PCL-5). Sociodemographics, clinical data, history of childhood trauma (Childhood Trauma Questionnaire [CTQ]), and exposure to potentially traumatic events (Life Events Checklist for DSM-5 [LEC-5]) were assessed.

**Results:**

Of the 778 ICU survivors included during the first wave of COVID-19 pandemic in France, 417 and 361 were assigned to the COVID-19 and non-COVID-19 cohorts, respectively. Fourteen (4.9%) and 11 (4.9%), respectively, presented with presumptive diagnosis of PTSD at 6 months (*p* = 0.976). After adjusting for age, sex, severity score at admission, use of invasive mechanical ventilation, ICU duration, CTQ and LEC-5, COVID-19 status was not associated with presumptive diagnosis of PTSD using the PCL-5. Only female sex was associated with presumptive diagnosis of PTSD. However, COVID-19 patients reported significantly more intrusion and avoidance symptoms than non-COVID patients (39% vs. 29%, *p* = 0.015 and 27% vs. 19%, *p* = 0.030), respectively. The median PCL-5 score was higher in the COVID-19 than non-COVID-19 cohort (9 [3, 20] vs. 4 [2, 16], *p* = 0.034).

**Conclusion:**

Admission to the ICU for COVID-19 was not associated with a higher prevalence of PTSD compared with admission for another cause during the first wave of the COVID-19 pandemic in France. However, intrusion and avoidance symptoms were more frequent in COVID-19 patients than in non-COVID-19 patients.

*Trial Registration*: Clinicaltrials.gov Identifier NCT03991611, registered on June 19, 2019.

## Background

Critically ill patients in intensive care units (ICUs) are exposed to stressful conditions and experience discomfort from multiple sources [1–6], such as the environment or related to care provided in the ICU, depending on the care organization and patient’s health status. This discomfort may have short- and long-term consequences for survivors of critical illness [7], such as various degrees of anxiety and/or depression [8–10] or posttraumatic stress disorder (PTSD) [11–13], which may affect patients’ quality of life [14–17], slow down the recovery process [18], and lead to increasing healthcare utilization and considerable associated costs [19]. PTSD has been the most investigated post-ICU psychiatric morbidity. A recent review of 48 studies found that PTSD symptoms may affect one in five ICU patients [20]. Other authors have focused on preexisting risk factors [21, 22] and early detection methods after a patient is discharged from the ICU [23].

The causes of PTSD may be linked to non-modifiable pre-ICU factors such as the patient’s history of depression or their experience of traumatic life events, which may have occurred as early as childhood and that are often unknown to caregivers working in the ICU, or are linked to potentially modifiable ICU factors in connection with the delivery of care, or treatments, or stressful environmental conditions [21, 22]. PTSD may also be linked to the critical illness leading to ICU stay. Many authors consider that the rates of PTSD after coronavirus disease 2019 (COVID-19) infection, particularly in patients hospitalized in the ICU for COVID-19, are higher than in critically ill patients hospitalized in the ICU before the pandemic [24–30]. In the context of the global pandemic, especially during the first wave (i.e. Spring 2020 in France), the evolution and contagiousness of the disease was not fully understood. Therefore, care organizations were greatly modified with much less attention paid to limiting stressful factors (restricted or forbidden family visitation, dramatic increase in the ICU beds leading to new inadequately trained staff, accumulation of working hours, recently created ICUs), making ICU stay a traumatic experience that sometimes resulted in the development of PTSD by patients and family members [31], compounded by the disease being highly publicized and associated with a high mortality [32, 33].

The aim of this study was to compare the prevalence of PTSD at 6 months in two populations of ICU survivors hospitalized during the first wave of the COVID-19 pandemic: one group of patients admitted to the ICU for COVID-19 and another group was admitted to the ICU for another reason, taking into account psychological pre-ICU factors.

## Methods

The PTSD-REA COVID study was part of the PTSD-REA study (a stepped wedge cluster randomized trial: Reducing PTSD after ICU discharge with the IPREA3 program), which was conducted during the first wave of the COVID-19 pandemic in France, a period during which the tested intervention of the PTSD-REA study (i.e., implementation of the IPREA3 program), was suspended due to major changes in the organization of critical care in France. The PTSD-REA COVID study was a prospective observational comparative cohort study of patients admitted to the ICU for COVID-19 or another reason during the same period. It was approved by the Institutional Review Board of Ile-de-France IV, Hôpital Saint-Louis (Paris, France), and is reported using the recommendations of the STROBE statement [34].

### ICUs and patients

Thirty-one ICUs participated in the PTSD-REA COVID study. Of the 18 ICUs that initially participated in the PTSD-REA study, 15 of them agreed to participate in the PTSD-REA COVID study and 16 new ICUs were recruited. The ICUs included 3 medical, 8 surgical, and 20 mixed medical–surgical adult ICUs, located at academic tertiary care hospitals or community hospitals. All patients aged ≥ 18 years, who were admitted to the ICU between March 1 and April 30, 2020, and survived an ICU stay of at least 3 calendar days, were eligible for inclusion in the study at the end of the ICU stay.

The exclusion criteria included patients who were unable or refused to provide informed consent, were under trusteeship or not affiliated with the French social security, were already included in the PTSD-REA study during a previous ICU stay, had cognitive incapacity, did not understand French sufficiently to complete questionnaires on psychiatric morbidity, or who transferred to another ICU at the end of the index ICU stay; collegial physician decision not to readmit to the ICU if the condition worsens after ICU discharge; patient’s advance directives in favor of no readmission to the ICU; or life expectancy of < 6 months at the end of the ICU stay. Consent was obtained from all participants.

According to the reason for admission, patients were assigned either to the COVID-19 cohort or the non-COVID-19 cohort. COVID-19 was diagnosed at hospital admission by positive real-time polymerase chain test or COVID-19 compatible or typical chest computed tomography pattern.

### Data collection and definition of PTSD symptoms

In each participating ICU, a trained research assistant was dedicated to collecting demographic and medical data including cause of admission and main life support therapies. Dedicated trained psychologists were specifically recruited outside the participating ICUs to conduct the telephone interviews and collect PTSD symptoms and other data at the 6-month post-ICU follow-up (e.g., anxiety and depressive symptoms, experience of traumatic events, and history of childhood trauma or maltreatment). Training sessions conducted by a psychiatrist with expertise in PTSD were organized to increase inter-rater reliability.

PTSD symptoms were assessed using the French version of the PTSD Checklist for Diagnostic and Statistical Manual of Mental Disorders (DSM-5) (PCL-5) [35], which aligns fully with the most recent version of the Diagnostic and Statistical Manual of Mental Disorders (DSM-5) [36]. The PCL-5 is a questionnaire consisting of 20 items that correspond to the DSM four-factor conceptualization of PTSD and its symptom clusters: intrusion symptoms (items 1 to 5), avoidance symptoms (items 6 to 7), negative alterations in cognition and mood (items 8 to 14), and increased arousal and reactivity (items 15 to 20). Thus, the PCL-5 thus contains four subscales corresponding to the abovementioned four symptom clusters. Each item is rated on a 5-point Likert-type scale from 0 (not at all) to 4 (extremely). The PCL-5 yields a total score ranging from 0 to 80, with higher scores indicating increased probability of having PTSD related to the event (i.e., index ICU hospitalization). In fact, all the patients were asked to complete the PCL-5 by indicating symptoms present in the past 30 days and related to their ICU experience.

The revised version of the Life Events Checklist for DSM-5 (LEC-5), which assesses exposure to 17 potentially traumatic events, was administered in addition to the PCL-5 to obtain a more exhaustive assessment of DSM-5 criterion A for PTSD [37]. The LEC-5 questionnaire was completed on a lifetime basis, enabling us to assess situations that increased the risk of PTSD prior to ICU hospitalization. For each event, the patient had to select the appropriate responses among the following proposals: (1) It happened to you personally; (2) you witnessed it happen to someone else; (3) you learned about I it happening to a close family member or close friend; (4) you were exposed to it as a part of your job; (5) you are not sure if it fits; or (6) it does not apply to you. Among the 17 potentially traumatic events, due to possible different impact of traumatic experiences on the occurrence of PTSD [38], we identified 4 items where a subject was victimized (victimization items) [39], namely physical assault, assault with a weapon, sexual assault, and other unwanted or uncomfortable sexual experience, which allowed us to determine whether each patient had been exposed to one or more of these four victimization items, either personally or as a witness.

A history of childhood trauma and maltreatment was assessed using the short version of the Childhood Trauma Questionnaire (CTQ), a 28-item questionnaire with a 5-point Likert-type scale (1 = “never true”, 2 = “rarely true”, 3 = “sometimes true”, 4 = “often true”, 5 = “very often true”). The CTQ is a retrospective measure of childhood trauma that has been psychometrically assessed in diverse populations [40]. Of the 28 items on the CTQ, 25 are split into five subscales: emotional abuse, physical abuse, sexual abuse, emotional neglect, and physical neglect. The three remaining items comprising the minimization/denial scale are used to determine if respondents are underreporting their childhood trauma. Each subscale is represented by questions with a score ranging from 5 to 25, which falls into four categories of trauma exposure intensity: none to low, low to moderate, moderate to severe, and severe to extreme. Scores above the recommended cut-off score for “low” severity on each of the subscales were considered cases of abuse and neglect. This criterion was chosen to emphasize test sensitivity to less severe cases.

We also assessed anxiety and depression symptoms at 6 months using the Hospital Anxiety and Depression Scale (HADS) [41]. The anxiety and depression subscales of HADS both include seven items, each rated on a 4-point scale from 0 to 3. Anxiety and depression scores yield scores from 0 to 21, with higher scores indicating increased probability of having anxiety or depression. Patients with a score ≥ 8 on either subscale are considered symptomatic with general anxiety or depressive symptoms [9, 10]. The HADS was developed to detect psychiatric symptoms in general medical patients [42].

### Outcome measures

All outcome measures pertained to the individuals. The primary outcome was the prevalence of substantial PTSD symptoms (i.e., presumptive diagnosis of PTSD) as assessed by the PCL-5 as follows: a cut-off total score ≥ 33 and at least one of re-experiencing or intrusion symptom, one avoidance symptom, two symptoms of negative alterations in cognition and mood, and two arousal symptoms. A symptom was considered present and clinically relevant with a score of 2 or above [43, 44]. Secondary outcomes were total score on the PCL-5, prevalence of at least one re-experiencing symptom, prevalence of at least one avoidance symptom, prevalence of at least two symptoms of negative alterations in cognition and mood, prevalence of at least two arousal symptoms, prevalence of substantial anxiety symptoms, HADS anxiety score, prevalence of depressive symptoms, HADS depression score and markers of healthcare consumption since ICU discharge (medical visit, emergency department visit, hospitalization, psychotropic drug use, psychologic and/or psychiatric care).

### Statistical analysis

Comparisons of baseline characteristics at ICU admission and during ICU stay between COVID-19 and non-COVID-19 patients were performed using the Student’s *t*-test or Mann–Whitney test for continuous variables (according to the variable distribution) and the chi-squared test or Fisher’s exact test for qualitative variables. The proportion of patients with PTSD and anxiety and depression symptomatology was provided for the whole sample and per group. Profiles of individuals with a presumptive diagnosis of PTSD, and substantial symptoms from each symptom cluster at 6 months after ICU discharge were determined. To identify the role of COVID-19 status in the presumptive diagnosis of PTSD and presence of substantial symptoms from each cluster symptom of PTSD, adjustment of eight confounding variables was performed using logistic regressions: age, sex, simplified acute physiology score (SAPS) II score, use of invasive mechanical ventilation, ICU duration, CTQ score, and exposure (either personally or as a witness) to at least one of the four victimization items on the LEC-5. The results are presented using odd ratios (OR) and their 95% confidence intervals (CIs). A sensitivity analysis was performed on the PCL-5 score as a dependent variable using a linear regression. The results are presented as standardized beta coefficients. All statistical analyses were performed with SPSS version 20.0 software.

## Results

### ICUs and patients

**Fig. 1:**
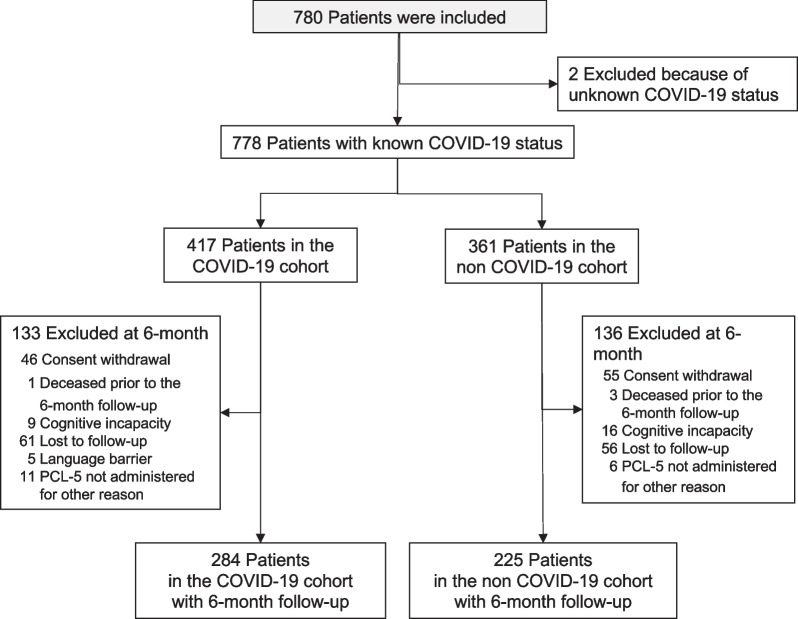
CONSORT-style flow diagram of patients in the PTSD-REA COVID study. *PCL-5* PTSD Checklist for DSM-5

Among the 31 participating ICUs in the PTSD-REA study, 13 of them are located at academic tertiary care hospitals, and 18 are located at community hospitals. This study followed patients who were hospitalized in these 31 ICUs between March and April 2020 for COVID-19 or another cause. A total of 780 ICU survivors were included. The COVID-19 status was unknown for two patients, and 417 patients were assigned to the COVID-19 cohort, and 361 to the non-COVID-19 cohort. A 6-month follow-up was conducted for 509 patients (284 and 225 in the COVID-19 and non-COVID-19 cohorts, respectively) (Fig. 1). Of the 778 survivors assigned to both cohorts, 4 (0.5%) died before the 6-month follow-up assessment, 117 (15.0%) were lost to follow-up, 166 (21.3%) withdrew consent when contacted by telephone by the psychologist, 25 (3.2%) had cognitive incapacity to answer self-reported questionnaires, and 5 (0.6%) had a language barrier (Fig. 1). Because of missing PCL-5, 283 and 225 ICU survivors were analyzed for the primary outcome in the COVID-19 and non-COVID-19 cohorts, respectively. Key clinical and demographic characteristics at ICU admission, and use of life support therapies are presented in Table 1. Patients in the COVID-19 cohort were more often treated with invasive mechanical ventilation, extracorporeal membrane oxygenation, and vasopressors, and had a longer ICU stay than patients in the non-COVID-19 cohort (Table 1).

**Table 1:** Baseline characteristics of patients according to COVID-19 status (*n* = 509 patients)

Variable	Total (*n* = 509)	COVID-19 (*n* = 284)	Non-COVID-19 (*n* = 225)	*p* value
Age, year, mean (SD)	62 (13)	63 (11)	60 (14)	0.073
Male, *n* (%)	329 (65)	191 (67%)	138 (61%)	0.165
SAPS II score^a^, mean (SD)	35.8 (14.9) [508]	35.5 (13.5) [283]	36.2 (16.6)	0.980
**Invasive mechanical ventilation, *n* (%)**	**304/508 (60)**	**202/283 (71)**	**102 (45)**	** < 0.01**
Non-invasive ventilation, *n* (%)	179/507 (35)	107/282 (38)	72 (32)	0.164
**High-flow nasal oxygen, *n* (%)**	**142/507 (28)**	**105/282 (37)**	**37 (16)**	** < 0.01**
**Extracorporeal membrane oxygenation, *n* (%)**	**22 (4)**	**17 (6)**	**5 (2)**	**0.038**
**Use of vasopressors, *n* (%)**	**240/507 (47)**	**151/282 (53)**	**89 (40)**	**0.02**
Renal replacement therapy, *n* (%)	42/507 (8)	23/282 (8)	19 (8)	0.907
**Days in ICU, median (interquartile range)**	**7 (4–16)**	**12 (6–25)**	**5 (3–8)**	** < 0.01**
Days in hospital after ICU discharge, median (interquartile range)	6 (2–12)	6 (0–13)	6 (3–12)	0.308

Boldface indicates significance with *p* < 0.05

*SAPS* Simplified Acute Physiology Score, *ICU* Intensive care unit

^a^SAPS II score may range from 0 to 156, with higher scores indicating more severe illness

### Psychiatric morbidity and healthcare consumption at 6 months after ICU discharge

Of the 508 patients with available PCL-5 at 6 months after ICU discharge, 25 patients (4.9%) presented with presumptive diagnosis of PTSD based on all required criteria derived from the PCL-5. The prevalence of presumptive diagnosis of PTSD as assessed by PCL-5 did not differ between COVID-19 and non-COVID patients. COVID-19 patients reported significantly more frequent intrusion and avoidance symptoms than non-COVID patients (39% vs. 29%, *p* = 0.015 and 27% vs. 19%, *p* = 0.030, respectively)*.* No significant difference was found between COVID and non-COVID patients with respect to the other symptom clusters. The median PCL-5 score was significantly higher in the COVID-19 cohort than in the non-COVID-19 cohort, 9 (3–20) vs. 7 (2–16) (*p* = 0.034) (Table 2). Of the 505 patients with available HADS, 154 (30%) and 86 (17%) patients presented with substantial anxiety and depression symptoms, respectively. The proportion of patients with substantial anxiety and depression symptoms did not significantly differ between COVID and non-COVID patients, as well as anxiety and depression scores derived from the HADS (Table 2).

**Table 2:** Posttraumatic stress disorder (PTSD), substantial anxiety and depression symptoms and healthcare consumption after ICU discharge at 6 months (*n* = 509 patients)

Variable	Total (*n* = 509)	COVID-19 (*n* = 284)	Non-COVID-19 (*n* = 225)	*p* value
*PTSD*				
Presumptive diagnosis of PTSD^a^, n (%)	25/508 (4.9)	14/283 (4.9)	11 (4.9)	0.976
**Intrusion symptoms, *n* (%)**	**176/508 (35)**	**111/283 (39)**	**65 (29)**	**0.015**
**Avoidance symptoms, *n* (%)**	**118/508 (23)**	**76/283 (27)**	**42 (19)**	**0.030**
Cognition and mood-related symptoms, *n* (%)	149/508 (29)	91/283 (32)	58 (26)	0.117
Arousal symptoms, *n* (%)	170/508 (33)	97/283 (34)	73 (32)	0.664
**PCL-5 total score**^**b**^	**7 (3–18)**	**9 (3–20)**	**7 (2–16)**	**0.033**
*Anxiety symptoms*				
HADS anxiety score^c^	5 (2–8)	5 (3–8)	5 (2–8)	0.372
Substantial anxiety symptoms^d^, *n* (%)	154/505 (30)	93/283 (33)	61/222 (27)	0.194
*Depression symptoms*				
HADS depression score^c^	3 (1–6)	3 (1–6)	3 (1–6)	0.973
Substantial depression symptoms^d^, *n* (%)	86/505 (17)	48/283 (17)	38/222 (17)	0.963
*Health consumption since ICU discharge*				
Visit to primary care physician, *n* (%)	466/506 (92)	260/283 (92)	206/223 (927)	0.835
**Emergency department visit, *n* (%)**	**73/501 (15)**	**33/281 (23)**	**40/220 (18)**	**0.043**
**Hospitalization, *n* (%)**	**138/501 (28)**	**66/281 (17)**	**72/222 (33)**	**0.022**
Psychotropic drug use, *n* (%)	78/503 (16)	46/280 (16)	32/223 (14)	0.522
Psychologic and /or psychiatric care, *n* (%)	58/503 (12)	31/280 (11)	27/223 (12)	0.718

Boldface indicates significance with *p* < 0.05

*ICU* Intensive care unit, *PCL-5* PTSD Checklist for DSM-5, *HADS* Hospital Anxiety and Depression Scale

PCL-5 total score, HADS anxiety score, and HADS depression score are presented as the median with interquartile range (IQR) in parentheses

^a^PTSD was diagnosed using the cut-off total score of 33 with at least one intrusion symptom, one avoidance symptom, two symptoms of negative alterations in cognition and mood, and two arousal symptoms. A symptom was considered present if the score of the corresponding item was 2 or above on a 5-point Likert-type scale

^b^PCL-5 score is a self-reported questionnaire consisted of 20 items; each related on a 5-point scale from 0 to 4 and may range from 0 to 80, with higher scores indicating increased probability of having PTSD

^c^The anxiety and depression subscales of HADS both include 7 items; each rated on a 4-point scale from 0 to 3 and may range from 0 to 21 with higher scores indicating increased probability of having anxiety or depression, respectively

^d^ Patients with a score ≥ 8 on each subscale were considered with substantial symptoms

During the 6 months following the ICU discharge, most patients (92%) visited their primary care physician, 15% went at least once to the emergency department, and 28% were hospitalized again at least once. Of the 503 patients assessed at 6 months with available data regarding psychological and/or psychiatric care, 16% reported continuous or intermittent use of psychotropic drugs, and 12% had benefited from psychologic and/or psychiatric care. Use of psychotropic drugs and psychologic and/or psychiatric care after ICU discharge were found to be linked with the presumptive diagnosis of PTSD in both cohorts.

### Impact of COVID-19 status on the presumptive diagnosis of PTSD at 6 months after ICU discharge

Univariate analysis showed that sex (female) and exposure to the victimization items on the LEC-5 were positively correlated, and age and ICU stay duration were negatively with the presumptive diagnosis of PTSD (Table 3). Through a multivariate model built with adjustment for the eight potential confounding covariates (age, sex, SAPS II score, use of invasive mechanical ventilation, ICU duration, history of childhood trauma and maltreatment as assessed by the total score derived from CTQ, and exposure to at least one of the four victimization items of the LEC-5), COVID-19 was not linked to the presumptive diagnosis of PTSD. Only female sex was found to be linked to the presumptive diagnosis of PTSD. The COVID-19 status was not linked to the PCL-5 score as a continuous variable (Table 4). The COVID-19 status was linked to the intrusion and avoidance symptoms, but not to symptoms of negative alterations in cognition and mood or arousal symptoms. For each of the four symptom clusters of PTSD, determinants among the eight potential confounding covariates are presented in Table 4.

**Table 3:** Determinants of presumptive diagnosis of PTSD: univariate analysis (*n* = 508 patients)

		No PTSD (*n* = 483)	PTSD (*n* = 25)	*p* value
COVID-19 status	No COVID-19, *n*(%)	214/483 (44%)	11/25 (44%)	0.976
	COVID-19, *n*(%)	269/483 (56%)	14/25 (56%)	
**Sex**	**Men, *n*(%)**	**323/483 (67%)**	**5/25 (20%)**	** < 0.001**
	**Women, *n*(%)**	**160/483 (33%)**	**20/25 (80%)**	
**Age, mean (SD)**		**62 (13) [483]**	**54 (15) **[25]	**0.001**
SAPS II^a^, mean (SD)		36 (15) [482]	31 (12) [25]	0.122
Invasive mechanical ventilation	No, *n*(%)	194/483 (40%)	10/24 (42%)	0.884
	Yes, *n*(%)	289/483 (60%)	14/24 (58%)	
Non-invasive ventilation	No, *n*(%)	309/482 (64%)	18/24 (75%)	0.276
	Yes, *n*(%)	173/482 (36%)	6/24 (25%)	
High-flow nasal oxygen	No, *n*(%)	346/482 (72%)	18/24 (75%)	0.732
	Yes, *n*(%)	136/482 (28%)	6/24 (25%)	
Use of vasopressors	No, *n*(%)	252/482 (52%)	15/24 (63%)	0.328
	Yes, *n*(%)	230/482 (48%)	9/24 (38%)	
**Days in ICU**		**7 (4–16) [483]**	**4 (3–9) **[25]	**0.046**
Days in hospital after ICU discharge		6 (2–12) [483]	5 (2–13)[25]	0.781
Childhood trauma and maltreatment (CTQ)^b^				
Emotional abuse	No, *n*(%)	269/333 (81%)	12/18 (67%)	0.220
	Yes, *n*(%)	64/333 (19%)	6/18 (33%)	
Physical abuse	No, *n*(%)	293/336 (87%)	13/18 (72%)	0.081
	Yes, *n*(%)	43/336 (13%)	5/18 (28%)	
Sexual abuse	No, *n*(%)	306/337 (91%)	14/18 (78%)	0.089
	Yes, *n*(%)	31/337 (9%)	4/18 (22%)	
Physical neglect	No, *n*(%)	277/336 (82%)	14/18 (78%)	0.539
	Yes, *n*(%)	59/336 (18%)	4/18 (22%)	
Emotional neglect	No, *n*(%)	186/337 (55%)	11/18 (61%)	0.809
	Yes, *n*(%)	151/337 (45%)	7/18 (39%)	
CTQ total score^c^		31 (28–37) [343]	36 (29–43) [18]	0.126
Exposure to the victimization items of LEC5^d^	No, *n*(%)	342/465 (74%)	11/24 (46%)	
	Yes, *n*(%)	123/465 (26%)	13/24 (54%)	0.003

Boldface indicates significance with *p* < 0.05

*PTSD* posttraumatic stress disorder, *SAPS* Simplified Acute Physiology Score, *CTQ* Childhood trauma questionnaire, *LEC-5*, Life Events Checklist for DSM-5

Days in ICU, days after ICU discharge, and CTQ total score are presented as median with interquartile range (IQR) in parentheses

^a^ SAPS II score may range from 0 to 156, with higher scores indicating more severe illness

^b^ The CTQ is a 28-item questionnaire with a 5-point Likert-type scale (1 = never true, 2 = rarely true, 3 = sometimes true, 4 = often true, 5 = very often true) split into five subscales exploring emotional abuse, physical abuse, sexual abuse, physical neglect, and emotional neglect, occurred during childhood. Each subscale is represented by five questions with a score range from 5 to 25. Guidelines specified the range of scores that fall into four categories according to the severity of abuse or neglect for each subscale: none to low, low to moderate, moderate to severe, and severe to extreme. Scores above the recommended cut-score for low severity on each of the subscales were considered cases of abuse and neglect. This criterion was chosen to emphasize test sensitivity to less severe cases

^c^We used in our model the sum of the 25 items exploring the five subscales (without taking into account the 3 minimization/denial items used in a scale that screens for the likelihood of underreporting traumatic experiences)

^d^We used in our model only exposure (as personally or as a witness) to one or more of the 4 items of victimization (physical assault, assault with a weapon, sexual assault or other unwanted or uncomfortable sexual experience) among the 17 items of the LEC-5

**Table 4:** Determinants of presumptive PTSD and each symptom cluster of PTSD at 6 months after ICU discharge: multivariate analysis (*n* = 508 patients)

	OR (95% CI)	*p* value
*Presumptive diagnosis of PTSD*^*a*^		
COVID-19 status	1.436 (0.480–4.297)	0.517
**Female sex**	**5.632 (1.722–18.424)**	**0.004**
Age	0.979 (0.943–1.016)	0.264
SAPS II^b^	0,988 (0.949–1.029)	0.553
Invasive mechanical ventilation	1.587 (0.513–4.907)	0.422
Days in the ICU	0.978 (0.925–1.034)	0.435
Childhood trauma as assessed by CTQ^c^	1.001 (0.965–1.039)	0.948
Exposure to potentially traumatic “victimization” events as assessed by LEC-5^d^	2.160 (0.736–6.343)	0.161
*Intrusion symptoms of PTSD*^*e*^		
**COVID-19 status**	**1.897 [1.113–3.234]**	**0.019**
**Female sex**	**2.812[1.716–4.608]**	** < 0.001**
**Age**	**0.974 [0.955–0.993]**	**0.007**
SAPS II^b^	0,998 [0.981–1.016]	0.842
Invasive mechanical ventilation	1.091 [0.625–1.904]	0.760
Days in the ICU	1.008 [0.992–1.025]	0.319
Childhood trauma and maltreatment as assessed by CTQ^c^	1.006 [0.985–1.028]	0.582
**Exposure to potentially traumatic “victimization” events as assessed by LEC-5**^**d**^	**1.909 [1.162–3.136]**	**0.011**
*Avoidance symptoms of PTSD*^*f*^		
**COVID-19 status**	**2.569 [1.386–4.763]**	**0.003**
**Female sex**	**2.464 [1.425–4.262]**	** < 0.001**
**Age**	**0.959 [0.938–0.980]**	**0.001**
SAPS II^b^	0,992 [0.971–1.013]	0.444
Invasive mechanical ventilation	1.052 [0.562–1.971]	0.874
Days in the ICU	0.984 [0.961–1.008]	0.191
Childhood trauma and maltreatment as assessed by CTQ^c^	0.998 [0.975–1.022]	0.883
Exposure to potentially traumatic “victimization” events as assessed by LEC-5^d^	1.255 [0.706–2.231]	0.438
*Symptoms of negative alterations in cognition and mood*^*g*^		
COVID-19 status	1.542 [0.864–2.751]	0.143
**Female sex**	**4.482 [2.640–7.611]**	** < 0.001**
**Age**	**0.979 [0.959–0.999]**	**0.042**
SAPS II^b^	0,996 [0.977–1.015]	0.649
Invasive mechanical ventilation	2.113 [0.562–1.971]	0.017
Days in the ICU	0.984 [1.141–3.914]	0.268
**Childhood trauma and maltreatment as assessed by CTQ**^**c**^	**1.033 [1.009–1.059]**	**0.008**
**Exposure to potentially traumatic “victimization” events as assessed by LEC-5**^**d**^	**1.803 [1.050–3.098]**	**0.033**
*Arousal symptoms*^*h*^		
COVID-19 status	1.095 [0.644–1.862]	0.738
**Female sex**	**2.869 [1.754–4.694]**	** < 0.001**
**Age**	**0.973 [0.954–0.992]**	**0.006**
SAPSII^b^	0,988 [0.970–1.006]	0.184
Invasive mechanical ventilation	1.064 [0.607–1.863]	0.829
Days in the ICU	1.004 [0.987–1.022]	0.623
**Childhood trauma and maltreatment as assessed by CTQ**^**c**^	**1.038 [1.014–1.062]**	**0.002**
Exposure to potentially traumatic “victimization” events as assessed by LEC-5^d^	0.906 [0.537–1.527]	0.711
*PCL-5 score*	Standardized beta coefficients	
COVID-19 status	0.095	0.071
**Female sex**	**0.307**	** < 0.001**
**Age**	− **0.205**	** < 0.001**
SAPSII^b^	− 0,028	0.602
Invasive mechanical ventilation	0.084	0.123
Days in the ICU	− 0.028	0.607
**Childhood trauma and maltreatment as assessed by CTQ**^**c**^	**0.176**	** < 0.001**
Exposure to potentially traumatic “victimization” events as assessed by LEC-5^d^	0.047	0.037

Boldface indicates significance with *p* < 0.05

*PTSD* posttraumatic stress disorder, *ICU* intensive care unit, *OR* odds ratio, *CI* confidence interval *SAPS* Simplified Acute Physiology Score, *CTQ* Childhood trauma questionnaire, *LEC-5* Life Events Checklist for DSM-5, *PCL-5* PTSD Checklist for DSM-5

^a^Presumptive diagnosis of PTSD was assessed from PCL-5 as follows: a cut-off total score of 33 or above and at least one re-experiencing or intrusion symptom, one avoidance symptoms, two symptoms of negative alterations in cognition and mood, and two arousal symptoms. A symptom was considered present and clinically relevant with a score of 2 or above

^b^SAPS II score may range from 0 to 156, with higher scores indicating more severe illness

^c^The CTQ is a 28-item questionnaire with a 5-point Likert-type scale (1 = never true, 2 = rarely true, 3 = sometimes true, 4 = often true, 5 = very often true) split into five subscales exploring emotional abuse, physical abuse, sexual abuse, physical neglect, and emotional neglect, occurred during childhood. Each subscale is represented by five questions with a score range from 5 to 25. We used in our model the sum of these 25 items (without taking into account the 3 minimization/denial items)

^d^We used in our model only exposure (as personally or as a witness) to one or more of the 4 “victimization” items (physical assault, assault with a weapon, sexual assault or other unwanted or uncomfortable sexual experience) among the 17 items of the LEC-5

^e^Intrusion symptoms were considered present if at least one intrusive symptom was reported with a score of 2 or above

^f^Avoidance symptoms were considered present if at least one avoidance symptom was reported with a score of 2 or above

^g^Symptoms of negative alterations in cognition and mood were considered present if at least two symptoms of negative alterations in cognition and mood were reported with a score of 2 or above

^h^Arousal symptoms were considered present if at least two arousal symptoms were reported with a score of 2 or above

## Discussion

In this prospective cohort study involving critically ill adult patients hospitalized in France in the ICU during the first wave of the COVID-19 pandemic, regardless of the reason for admission (COVID-19 or another cause), and surviving an ICU hospitalization of 3 days or more, we found that the prevalence of presumptive diagnosis of PTSD based on all required criteria derived from the PCL-5 was low at about 5% and not impacted by COVID-19 status.

Regarding PTSD, we found a lower prevalence in both cohorts compared with previous studies [20, 22, 45, 46]. The use of restrictive criteria derived from the PCL-5 questionnaire for the presumptive diagnosis of PTSD could explain this difference. In fact, in most other studies, the authors used different diagnostic tools [20, 46], such as the revised impact of event scale (IES-R), which was not adapted to the new criteria for the presumptive diagnosis of PTSD according to the DSM-5. Another possible explanation for this difference is that we assessed PTSD only at a single time point, 6 months after ICU discharge, without being able to detect early-onset and rapidly improving PTSD or PTSD with a later onset.

Previous studies have shown that patients affected by COVID-19 should be considered as at risk patients for developing PTSD, and some authors have requested a specific survey for these patients to detect the occurrence of posttraumatic symptoms as early as possible [47]. We did not find these results for the presumptive diagnosis of PTSD assessed by PCL-5. However, we found differences in the prevalence of both intrusion and avoidance symptoms in patients hospitalized in the ICU for COVID-19 compared with ICU hospitalization for other causes. Moreover, the total score on the PCL-5 was higher in the COVID-19 than non-COVID-19 cohort. These results and the fact that intrusion symptoms are central to the diagnosis of PTSD suggest that COVID-19 infection resulting in ICU stay could have an impact on the occurrence of PTSD.

Risk factors for PTSD after an ICU stay can be classified as pre-ICU non-modifiable factors (psychological factors such as depression history or previous PTSD, and demographic factors such as age and sex) and factors linked to the ICU hospitalization such as the actual cause or syndrome leading to ICU stay, life support therapies implemented in the ICU, perception of threat to life, delusional memories, delirium, or other factors making the ICU experience more traumatic [22]. In our study, as might be expected given the results of epidemiologic COVID-19 studies [32], characteristics of ICU stays for COVID-19 were different from those of ICU stays for other reasons: the patients affected by COVID-19, most of whom having presented with pneumonia characterized by severe hypoxemia, were much more often treated with invasive mechanical ventilation and had a longer ICU stay than patients admitted to the ICU for causes other than COVID-19. Finally, COVID-19 affected men more often than women and the age distribution in this study was specific for COVID-19 with a higher incidence in older patients. Therefore, it was essential for our multivariate model to take into account these differences, namely age, sex, severity score, use of invasive mechanical ventilation, and ICU duration, to assess the impact of COVID-19 on the occurrence of PTSD.

Our study had significant methodological strengths. First, to the best of our knowledge, this study is the first prospective cohort study comparing critically ill patients hospitalized in the same ICU during the same period whether for COVID-19 or another cause. The impact of being hospitalized in the ICU during the pandemic period, a time in which normal ICU operations were disrupted (most often by banning visits from relatives) and characterized by intensive and daily media coverage, was the same for both cohorts, making it possible to compare the impact of PTSD according to COVID-19 status without bias. Second, although we did not collect precise data on psychiatric history, usually considered as a risk factor for PTSD, we collected other pre-ICU psychological risk factors for PTSD and introduced previous trauma experiences in our multivariate model, as assessed by the administration of LEC-5 and even childhood trauma or maltreatment during the childhood. To the best of our knowledge, this is the only study involving critically ill patients taking into account such early psychological non-modifiable factors. Third, unlike other authors who have used previous questionnaires to detect PTSD such as IES-R, which do not take into account the new fourth symptom cluster of negative alterations in cognition and mood included in the actualized diagnostic of PTSD according to the DSM-5, we used the PCL-5 for the presumptive diagnosis of PTSD that is fully aligned with the DSM-5, even if the PCL-5 has not been yet validated in ICU populations [48]. Fourth, trained psychologists who administered PCL-5 were independent from all the participating ICUs and were unaware of the reason for ICU admission.

Our study also had several limitations. First, we assessed PTSD symptoms only at 6 months, making impossible to detect the evolution of eventual PTSD symptoms by using several follow-up time points: either late PTSD symptoms beyond 6 months, or early PTSD symptoms resolved before the 6-month follow-up. Second, our study only assessed the presumptive diagnosis of PTSD and was not designed to confirm the diagnosis of PTSD with a semi-structured PTSD clinical interview, which is considered the gold-standard tool to diagnose PTSD [49]. Third, the response rate was relatively low with about one-third of included patients not assessed with PCL-5 at 6 months. However, our response rate was higher than the response rates reported by authors conducting postal surveys to detect depression, anxiety, or PTSD symptoms after an ICU stay [45]. Moreover, the primary objective of our study was to compare the prevalence of PTSD in critically ill COVID-19 and non-COVID-19 patients, and the response rate was similar in both cohorts making our conclusion valid. Fourth, the assessments were only telephone-based, which is usually reliable enough for data collection as compared to in-person interviews, but face-to-face interviews may be preferred by some patients. Nonetheless, teleconsultation was the preferred option during the pandemic. Fifth, we did not perform formal inter-rater reliability assessments of our psychologists who conducted the telephone-based assessments, which could affect our findings. However, all of the psychologists were highly trained in administering mental health measures and received clear instructions to harmonize the evaluation practice. Sixthly, we excluded patients with low probability of survival at 6 months as well as those considered a priori unable to completing the PCL-5 using the PCL-5. These exclusion criteria could have altered the prevalence of PTSD at 6 months. These limitations combined with the use of PCL-5 may explain the lower prevalence of PTSD after an ICU stay than that reported by most authors. Another limitation of our study was the lack of precise data characterizing the ICU stay, especially data related to potential painful or discomfort-inducing procedures, as well as the presence of delirium or hallucinations, which may promote PTSD [50]. We did not collect data regarding sedative drugs, especially the use of benzodiazepines [22] or the use of physical restraints [51] considered by some authors as a risk factor for PTSD or glucocorticoids, which could have protective effects on the onset of PTSD after ICU stay [52]. We also did not measure the overall discomfort score at the end of the ICU stay, which may influence the occurrence of PTSD after ICU stay [53]. We did not explore in depth the nature of intrusive memories in patients with intrusion symptoms, whether they were hallucinations and/or delusions, or factual events from the ICU, since frightening hallucinations/delusions are considered to increase the risk of PTSD than memories of real events [50]. Finally, since we collected the CTQ and the LEC-5 several months after ICU discharge, we may have underestimated the incidence of these traumatic events that may have occurred prior to ICU stay, due to possible long-term cognitive impairment after critical illness [7]. In this respect, we recommend in future studies to use life history calendars to improve recall of potentially traumatic experience [54].

Our study confirmed the central role of sex as a risk factor for PTSD. Similar to other authors, we did not find an association between the severity of the critical illness at admission to the ICU, and ICU duration or invasive mechanical ventilation [22]. Non-modifiable pre-ICU risk factors such as sex and to a lesser extent age, or preexisting psychologic symptoms or psychiatric history seem to play a more important role in the occurrence of ICU-related PTSD than modifiable in ICU factors [21]. Even if COVID-19 status was not found to be an independent variable associated with the prevalence of PTSD at 6 months after ICU discharge, some diagnosis clusters of PTSD were more frequent at 6 months in COVID-19 patients than in non-COVID-19 patients.

In conclusion, the COVID-19 status was not associated with PTSD as assessed by PCL-5 at 6 months after ICU discharge. However, being hospitalized in the ICU for COVID-19 seems to lead to more frequent intrusion and avoidance symptoms, which correspond to the first two diagnosis clusters of PTSD.

## Data Availability

The datasets used and analyzed during the current study are available from the corresponding author in response to reasonable requests.

## Abbreviations

COVID-19: Coronavirus disease 2019
CTQ: Childhood trauma questionnaire
ICU: Intensive care unit
LEC-5: Life events checklist for DSM-5
PCL-5: PTSD Checklist for DSM-5
PTSD: Posttraumatic stress disorder
